# Case Report: Necrotizing myositis secondary to a pararectal fistula: diagnostic challenges and multidisciplinary management

**DOI:** 10.3389/fmed.2026.1906467

**Published:** 2026-07-17

**Authors:** Andrea Neville Cracchiolo, Daniela Maria Palma, Marco Palmeri, Bruno Cracchiolo, Dario Nicosia, Fabio Genco, Luigi Profera, Santi Maurizio Raineri, Giuseppe Accurso

**Affiliations:** 1 Unit of 118 Emergency Medical Services and Hyperbaric Medicine, ARNAS Civico Di Cristina Benfratelli Hospital, Palermo, Sicily, Italy; 2 Department of Neuroscience and Emergency with Trauma Center, A.O.O.R. Villa Sofia Cervello, Palermo, Italy; 3 Vita-Salute San Raffaele University, Milan, Italy; 4 Department of Anaesthesia, Intensive Care and Emergency, Policlinico Paolo Giaccone, Palermo, Italy; 5 Department of Precision Medicine Disciplines in the Medical Area Surgical and Critical Care, University of Palermo, Palermo, Italy

**Keywords:** Group A Streptococcus, hyperbaric oxygen therapy, necrotizing myositis, necrotizing soft tissue infection, pararectal fistula

## Abstract

**Background:**

Necrotizing soft tissue infections (NSTIs) are rare but life-threatening conditions requiring early diagnosis and aggressive multidisciplinary management. Necrotizing myositis (NM) is an uncommon and particularly severe manifestation, often associated with diagnostic delay and poor outcomes.

**Case presentation:**

We report the case of a 57-year-old man presenting with mild pelvic pain radiating to the posterior thigh. Contrast-enhanced computed tomography revealed a presacral abscess with an extrasphincteric fistulous tract extending into the posterior thigh musculature. Surgical exploration confirmed necrotizing fasciitis with myonecrosis caused by Group A Streptococcus. The patient underwent repeated surgical debridement, broad-spectrum antimicrobial therapy, and adjunctive hyperbaric oxygen therapy, resulting in progressive clinical improvement and complete recovery at six-month follow-up.

**Conclusion:**

This case highlights the importance of early imaging, prompt source control, and multidisciplinary management in atypical NSTIs, while illustrating the potential adjunctive role of HBO_2_ in selected patients.

## Highlights

- Necrotizing myositis may originate from occult pararectal fistulas with atypical anatomical spread.
- Early imaging is crucial for identifying deep infectious pathways and guiding surgical management.
- Surgical debridement and broad-spectrum antibiotics remain the cornerstone of NSTI treatment.
- HBO_2_ may represent a useful adjunctive therapy in selected patients with extensive hypoxic soft tissue infections.
- Evidence supporting HBO_2_ in NSTIs remains limited, and further studies are needed.

## Introduction

Skin and soft tissue infections (SSTIs) encompass a broad spectrum of clinical conditions ranging from mild, self-limiting infections to rapidly progressive and life-threatening diseases. Among these, necrotizing soft tissue infections (NSTIs) represent a rare but severe subset characterized by rapid tissue destruction, systemic toxicity, and high mortality, particularly when diagnosis and treatment are delayed (1–3).

Necrotizing myositis (NM) is an uncommon and particularly aggressive form of NSTI involving the muscular compartment. It may occur as a primary infection or, more rarely, as a secondary process due to contiguous spread from adjacent anatomical structures (1, 4). Early recognition is often challenging, as initial clinical manifestations may be subtle and nonspecific, frequently leading to delays in diagnosis (5).

Prompt surgical intervention combined with broad-spectrum antimicrobial therapy remains the cornerstone of management (5, 6). However, the optimal role of adjunctive therapies, including hyperbaric oxygen therapy (HBO_2_), remains a matter of debate. HBO_2_ has been proposed to improve tissue oxygenation and host immune response in hypoxic infected tissues, although robust clinical evidence supporting its routine use is still limited (7, 8).

We report a rare case of necrotizing myositis secondary to a pararectal fistula with atypical extension to the posterior thigh. This case highlights the diagnostic challenges associated with unusual anatomical spread and provides clinical insight into the management of severe soft tissue infections, including the potential role of adjunctive HBO_2_.

## Case presentation

A 57-year-old male presented to the emergency department in March 2025 with mild lower abdominal pain radiating to the groin and posterior aspect of the left thigh, associated with difficulty walking.

His medical history was significant for rectal cancer treated 8 years earlier with surgery and radiotherapy. He had no other relevant comorbidities.

On admission, the patient was hemodynamically stable and afebrile. Physical examination revealed mild tenderness in the left groin and swelling of the posterior left thigh, with warm, erythematous, and tense skin.

Laboratory tests showed elevated inflammatory markers, including CRP (18 mg/dL), procalcitonin (13.84 ng/mL), increased creatine phosphokinase, and elevated myoglobin levels.

Ultrasound demonstrated muscle fiber disruption with edema. Contrast-enhanced CT identified a presacral collection with gas formation and an extra-sphincteric fistulous tract extending through the sacrotuberous ligament into the posterior thigh musculature (Figures 1, 2). These findings were consistent with necrotizing infection with muscular involvement.

**Figure 1:**
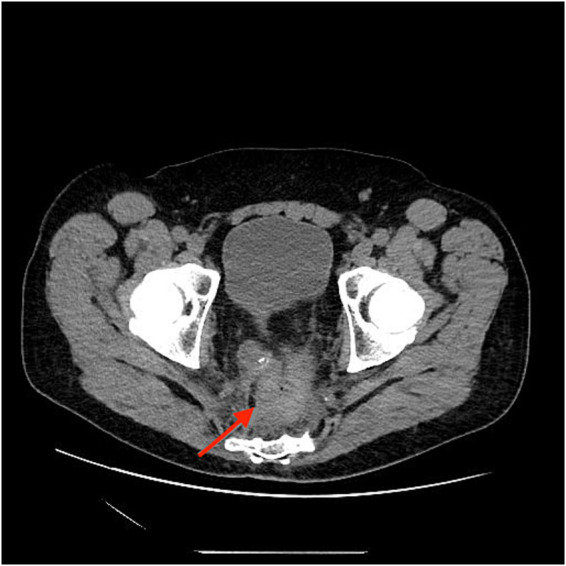
Contrast-enhanced CT scan showing a presacral collection with gas formation.

**Figure 2:**
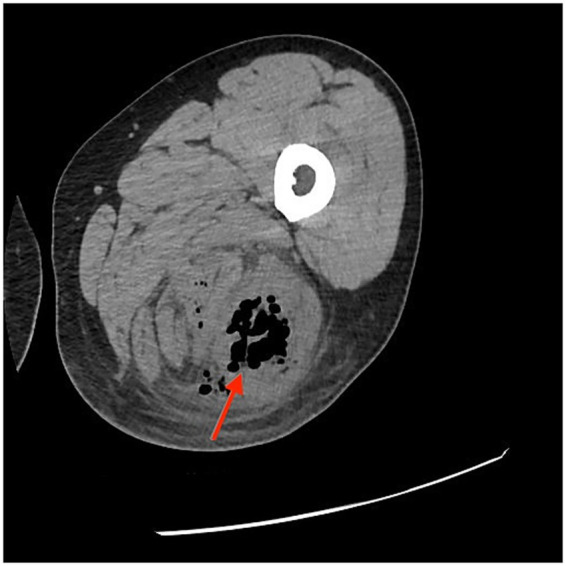
Contrast-enhanced CT scan of the left thigh demonstrating intramuscular gas and soft tissue involvement consistent with necrotizing infection.

Empirical intravenous antibiotic therapy with meropenem and linezolid was initiated. The patient was managed with a multidisciplinary approach involving surgeons, intensivists, and hyperbaric medicine specialists. Colonoscopy revealed a diverticular lesion communicating with the abscess cavity. No endoscopic or radiological findings suggestive of local rectal cancer recurrence were identified during the diagnostic work-up, supporting an inflammatory rather than neoplastic origin of the pararectal fistulous tract.

The patient underwent urgent surgical debridement, which revealed extensive necrotic tissue requiring fasciotomy and excision. A sigmoidostomy and fistulectomy were performed. Microbiological cultures grew Group A Streptococcus (Figure 3).

**Figure 3:**
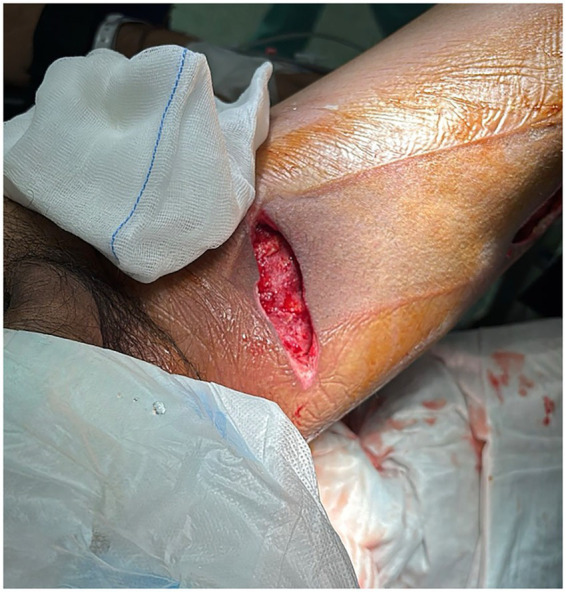
Postoperative clinical appearance after surgical debridement, showing viable tissue and absence of necrotizing changes.

Two further surgical revisions were required. Adjunctive HBO_2_ was initiated on postoperative day 1, consisting of daily sessions at 2.5–2.8 ATA for a total of 26 treatments. The therapy was well tolerated, with no adverse events (Figure 4).

**Figure 4:**
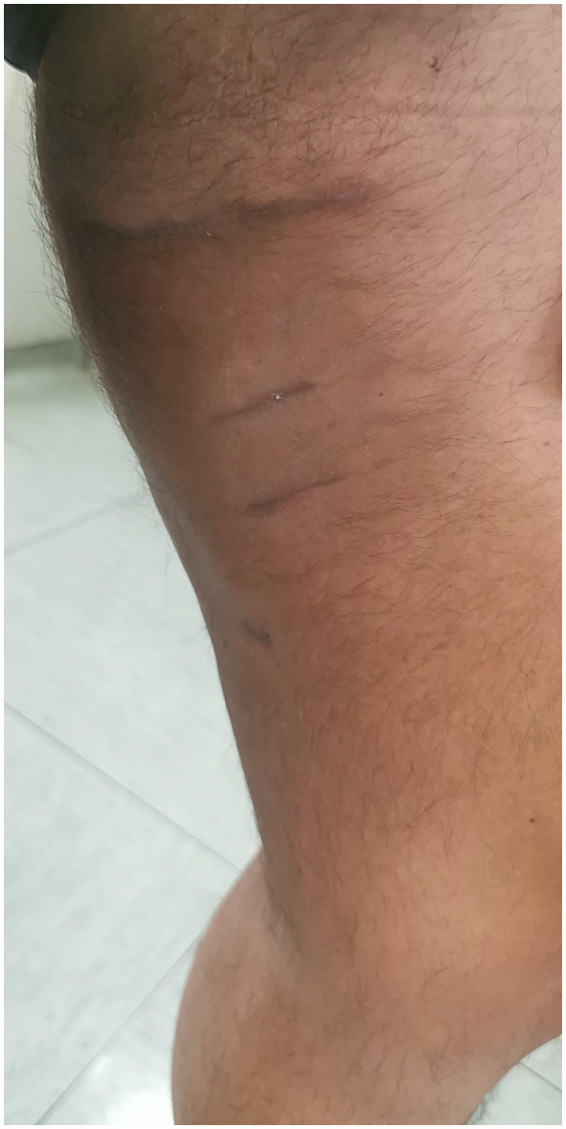
Clinical follow-up showing complete recovery (*restitutio ad integrum*) of the affected area.

Progressive clinical and biochemical improvement was observed. The patient was discharged after 4 weeks and achieved complete recovery at six-month follow-up (Table 1).

**Table 1:** Laboratory parameters and LRINEC score evolution.

Laboratory parameter	Pre HBO_2_	Post HBO_2_	Follow up
CRP	18 mg/dL	1.23 mg/dL	<0.5 mg/dL
WBC	9.4×10^3^/mm^3^	5.60×10^3^/mm^3^	5.35×10^3^/mm^3^
Hb	10.5 gr/dl	11.7 gr/dl	13.0 gr/dl
Na	135 mmol/L	141 mmol/L	139 mmol/L
Creat	0.90 mg/dL	0.85 mg/dL	0.79 mg/dL
BGluc	123 mg/dL	92 mg/dL	79 mg/dl
LRINEC	6	1	1

CRP, C-reactive protein; WBC, white blood cells; Hb, hemoglobin; Na, sodium; Creat, creatinine; BGluc, blood glucose; LRINEC, Laboratory Risk Indicator for Necrotizing Fasciitis. Pre HBO₂: admission; Post HBO₂: completion of HBO₂ treatment before discharge; Follow-up: six-month outpatient evaluation.

## Discussion

NSTIs are uncommon but severe infections characterized by rapid progression, extensive tissue destruction, and significant mortality, reported between 14 and 20% despite advances in management (1, 3, 9). Necrotizing myositis represents a rare and particularly aggressive form, often associated with delayed diagnosis and worse outcomes (9).

Based on microbiological features, NSTIs are broadly classified into polymicrobial and monomicrobial forms, the latter frequently caused by Group A Streptococcus (GAS) (10, 11). GAS infections are characterized by toxin-mediated tissue damage and immune dysregulation, leading to microvascular thrombosis, ischemia, and rapid necrosis (4).

Unlike more common NSTI presentations arising from skin trauma, surgical wounds, or perineal infections, the present case is exceptional because the necrotizing myositis originated from a pararectal diverticular abscess and extended to the posterior thigh through an extrasphincteric fistulous tract.

Empirical antibiotic therapy in NSTIs should be initiated promptly and provide broad-spectrum coverage against Gram-positive, Gram-negative, and anaerobic pathogens, particularly in infections arising from abdominal or perineal sources. Community-acquired NSTIs are commonly associated with Group A Streptococcus, *Staphylococcus aureus*, and polymicrobial flora, whereas in healthcare-associated settings, antimicrobial selection may need to consider resistant Gram-negative pathogens, including *Pseudomonas aeruginosa*, for which evolving therapeutic strategies have been recently described (10, 12, 13). Recommended empirical regimens typically include a broad-spectrum *β*-lactam agent, such as piperacillin/tazobactam or a carbapenem, combined with a protein synthesis inhibitor (e.g., clindamycin or linezolid) in suspected streptococcal infections to mitigate toxin production (6). In our case, the initial use of meropenem plus linezolid ensured adequate coverage and was subsequently supported by microbiological identification of Group A Streptococcus, confirming appropriate targeted therapy.

The present case is exceptional because the necrotizing myositis originated from a pararectal diverticular abscess and extended through an extrasphincteric fistulous tract to the posterior thigh, an atypical anatomical dissemination that highlights the importance of considering deep pelvic sources in patients presenting with thigh pain and soft tissue infection (14).

An additional factor that may have contributed to the development and extension of the infection is the patient’s history of pelvic radiotherapy for rectal cancer. Radiation-induced tissue injury is characterized by progressive fibrosis, microvascular damage, chronic hypoxia, and impaired local immune response, all of which may predispose to fistula formation, delayed tissue healing, and severe infectious complications. Although a direct causal relationship cannot be established, prior radiotherapy may have facilitated both the formation of the extrasphincteric fistulous tract and the extensive dissemination of the infection. Local tumor recurrence was excluded during the diagnostic work-up, supporting an inflammatory rather than neoplastic origin of the lesion. Interestingly, these pathophysiological mechanisms also provide a biological rationale for the use of adjunctive HBO₂, which is an established therapeutic option for radiation-induced soft tissue injury (15).

Early diagnosis remains challenging, as initial clinical findings may be nonspecific. Disproportionate pain represents a key clinical warning sign (5). Although the LRINEC score may assist in risk stratification, it lacks sufficient sensitivity to exclude NSTIs (16).

In this case, contrast-enhanced CT was pivotal because it simultaneously identified the presacral collection, demonstrated the extrasphincteric fistulous tract, and defined the extension to the posterior thigh musculature, thereby directly informing the surgical approach and source-control strategy (12).

Prompt and aggressive surgical debridement remains the cornerstone of treatment and is strongly associated with improved outcomes. In many cases, repeated surgical exploration is required to achieve adequate source control, as observed in our patient (4).

Adverse prognostic factors in NSTIs include delayed diagnosis, septic shock, organ dysfunction, extensive tissue or muscle involvement, and inadequate early source control. In the present case, the favorable outcome may be explained by early radiological diagnosis, hemodynamic stability at presentation, prompt surgical debridement, appropriate antimicrobial therapy, and coordinated multidisciplinary management (1, 9).

HBO_2_ has been proposed as an adjunctive treatment in NSTIs, particularly in cases characterized by extensive tissue hypoxia. Its potential benefits include increased tissue oxygenation, enhancement of leukocyte bactericidal activity, inhibition of anaerobic bacterial growth, and improved antibiotic efficacy (7, 17). In addition, HBO_2_ may contribute to tissue repair by modulating inflammatory responses and improving microcirculatory function (8, 18). In the present case, HBO₂ was initiated early in the postoperative course and was well tolerated, with no treatment-related adverse events, consistent with the favorable safety profile reported in previous studies (19). Although causality cannot be established, HBO₂ may have contributed to infection control and tissue recovery in the setting of extensive tissue hypoxia (8, 17).

However, clinical evidence supporting HBO_2_ remains limited. Most available data derive from observational studies and registry analyses, which suggest a potential reduction in mortality and surgical burden but are subject to selection bias. Systematic reviews have failed to provide definitive conclusions, and the routine use of HBO_2_ remains controversial (7, 8).

Therefore, despite a strong physiological rationale, HBO₂ should currently be considered an adjunctive therapy in selected patients rather than a routinely recommended intervention.

## Conclusion

Necrotizing soft tissue infections require early recognition and prompt multidisciplinary management to reduce morbidity and mortality. This case highlights a rare anatomical pathway of infection spread from a pararectal source to the posterior thigh, emphasizing the importance of maintaining a high index of suspicion and the pivotal role of imaging in identifying atypical presentations.

In particular, this case illustrates a diagnostic pitfall in which mild and nonspecific symptoms masked a severe deep-seated infection originating from a pelvic source. Early imaging played a crucial role in identifying the atypical spread and preventing further clinical deterioration, underscoring the importance of prompt radiological evaluation in patients presenting with disproportionate pain and minimal initial findings.

While early and aggressive surgical debridement remains the cornerstone of treatment, adjunctive strategies may play a role in selected patients. In this context, hyperbaric oxygen therapy may represent a useful supportive tool in severe infections characterized by extensive tissue hypoxia. However, given the limited and heterogeneous clinical evidence, its routine use remains controversial (7, 8, 17). Further prospective studies are warranted to better define patient selection and clarify the clinical impact of HBO_2_ in necrotizing soft tissue infections.

## Data Availability

The raw data supporting the conclusions of this article will be made available by the authors, without undue reservation.
